# Highlight selection of radiochemistry and radiopharmacy developments by editorial board

**DOI:** 10.1186/s41181-022-00177-w

**Published:** 2022-10-01

**Authors:** Jun Toyohara, Mohammed Al-Qahtani, Ya-Yao Huang, Emiliano Cazzola, Sergio Todde, Shozo Furumoto, Renata Mikolajczak, Clemens Decristoforo, Nic Gillings, Min Yang, Raymond Reilly, Adriano Duatti, Antonia Denkova, Ralf Schirrmacher, Giuseppe Carlucci, Yann Seimbille, Zhaofei Liu, Beverley Ellis, Bart T. Cornelissen, Klaus Kopka, Emerson Bernardes

**Affiliations:** 1 PET Radiopharmaceutical Sciences, Research Team for Neuroimaging, Tokyo Metropolitan Institute of Gerontology, Tokyo, Japan; 2 Cyclotron and Radiopharmaceuticals Department, King Faisal Specialist Hospital and Research Center (KFSHRC), Riyadh, Saudi Arabia; 3 Molecular Imaging Center, National Taiwan University, Taipei, Taiwan; 4 Scientific Institute for Research, Hospitalization and Healthcare (IRCCS), Sacro Cuore-Don Calabria Hospital, Negrar (Vr), Italy; 5 Istituto di Ricovero e Cura a Carattere Scientifico (IRCCS), Ospedale Sacro Cuore-Don Calabria, Negrar (Vr), Italia; 6 University of Milano-Bicocca, Tecnomed Foundation, Monza, Italy; 7 Cyclotron and Radioisotope Center (CYRIC), Tohoku University, Sendai, Japan; 8 Radioisotope Centre POLATOM, National Centre for Nuclear Research (NCBJ), Otwock, Poland; 9 Ośrodek Radioizotopów POLATOM, NARODOWE CENTRUM BADAŃ JĄDROWYCH (NCBJ), Otwock, Poland; 10 Department of Nuclear Medicine, Medical University of Innsbruck, Innsbruck, Austria; 11 Department of Clinical Physiology and Nuclear Medicine, Copenhagen University Hospital Rigshospitalet, Copenhagen, Denmark; 12 NHC Key Laboratory of Nuclear Medicine, Jiangsu Institute of Nuclear Medicine, Wuxi, Jiangsu China; 13 Department of Pharmaceutical Sciences, Leslie Dan Faculty of Pharmacy, University of Toronto, Toronto, ON Canada; 14 Department of Chemical, Pharmaceutical and Agricultural Sciences, University of Ferrara, Ferrara, Italy; 15 Dipartimento di Scienze Chimiche, Farmaceutiche e Agrarie Università di Ferrara, Ferrara, Italia; 16 Department of Radiation Science and Technology, Delft University of Technology, Delft, The Netherlands; 17 Department of Oncology, Cross Cancer Institute, University of Alberta, Edmonton, AB Canada; 18 Department of Molecular and Medical Pharmacology, University of California, Los Angeles (UCLA), Los Angeles, CA USA; 19 Department of Radiology and Nuclear Medicine, Erasmus MC, University Medical Center Rotterdam, Rotterdam, The Netherlands; 20 Medical Isotopes Research Center, Department of Radiation Medicine, School of Basic Medical Sciences, Peking University Health Science Centre, Beijing, China; 21 Manchester University NHS Foundation Trust, Manchester, UK; 22 Department of Nuclear Medicine and Molecular Imaging, Groningen (UMCG), University Medical Center, University of Groningen, Groningen, The Netherlands; 23 Helmholtz-Zentrum Dresden-Rossendorf (HZDR), Institute of Radiopharmaceutical Cancer Research, Dresden, Germany; 24 Energy and Nuclear Research Institute (IPEN-CNEN/SP), Cidade Universitária, São Paulo, Brazil; 25 Instituto de Pesquisas Energéticas e Nucleares (IPEN-CNEN/SP), Cidade Universitária, São Paulo, Brasil

**Keywords:** Highlight Articles, Radiochemistry, Radiopharmacy, Radiopharmaceutical Sciences, Nuclear Medicine, Trends in Radiopharmaceutical Sciences

## Abstract

**Background:**

The Editorial Board of EJNMMI Radiopharmacy and Chemistry releases a biannual highlight commentary to update the readership on trends in the field of radiopharmaceutical development.

**Main Body:**

This commentary of highlights has resulted in 21 different topics selected by each coauthoring Editorial Board member addressing a variety of aspects ranging from novel radiochemistry to first in man application of novel radiopharmaceuticals.

**Conclusion:**

Trends in radiochemistry and radiopharmacy are highlighted demonstrating the progress in the research field in various topics including new PET-labelling methods, FAPI-tracers and imaging, and radionuclide therapy being the scope of EJNMMI Radiopharmacy and Chemistry.

## Background

Each individual coauthoring member of the Editorial Board has selected a highlight article that has appeared in the radiochemistry, radiopharmacy and imaging agent literature during the period January-June 2022. The aim of this collaborative initiative is to create a biyearly overview for the readers summarizing the latest trends in the field.

## On-column conversion of [^11^C]methyl iodide to hydrogen [^11^C]cyanide without any special equipment and reagents

### By Jun Toyohara

**Fig. 1:**
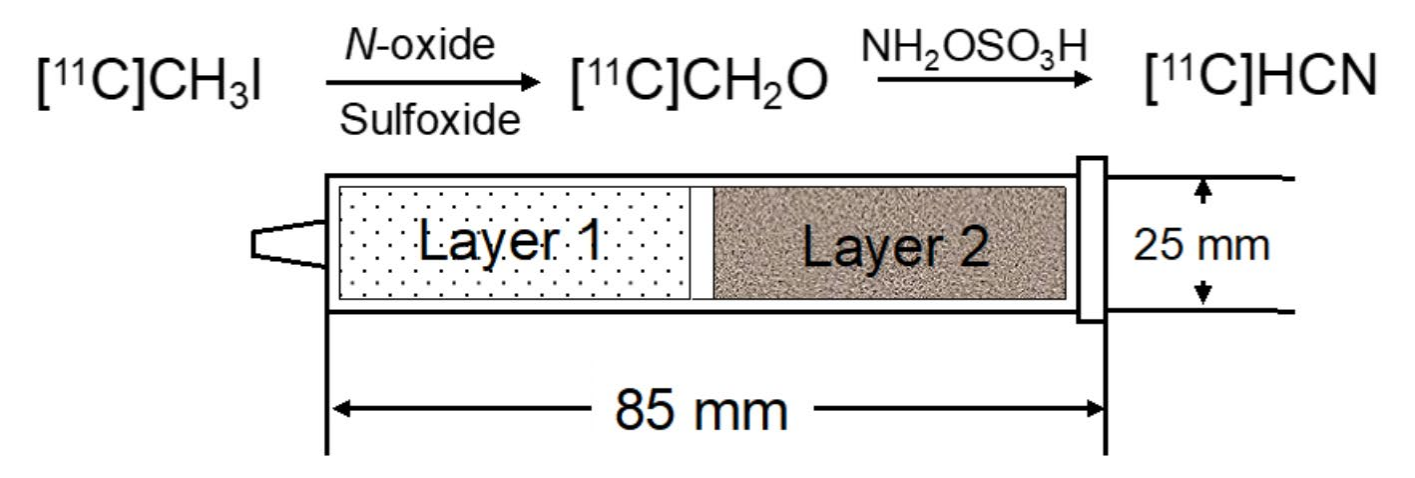
On-column conversion of [^11^C]CH_3_I to [^11^C]HCN (with permission from CCC Marketplace)

Carbon-11 cyanation with hydrogen [^11^C]cyanide ([^11^C]HCN) has been used to prepare a variety of ^11^C-labelled compounds. Furthermore, cyano groups are frequently introduced to pharmaceuticals, because they often improve the pharmacokinetics and pharmacodynamics of molecules. As such, the use of [^11^C]HCN is increasingly demanded in the field of radiopharmaceutical chemistry. However, current [^11^C]HCN production requires dedicated instruments, and is not as robust and reproducible as the production of [^11^C]methyl iodide ([^11^C]CH_3_I). Therefore, only a few facilities produce [^11^C]HCN, and compounds labelled with [^11^C]HCN are not widely used despite their usefulness. To make [^11^C]HCN more accessible, a simple on-column method was developed for the preparation of [^11^C]HCN from [^11^C]CH_3_I *via* [^11^C]formaldehyde ([^11^C]CH_2_O) (Kikuchi et al. 2022). Gaseous [^11^C]CH_3_I was simply passed through a 150 to 170 °C heated small glass two-layered reaction column. An instructional video on how to prepare the reaction column is available at https://www.rsc.org/suppdata/d1/sc/d1sc07033a/d1sc07033a1.mp4. The first layer in the small glass column, comprising *N*-oxide (oxymatrine) and sulfoxide (diphenyl sulfoxide)-soaked quartz glass wool, converted [^11^C]CH_3_I to [^11^C]CH_2_O, and the second layer, comprising a mixture of hydroxylamine-*O*-sulfoxide and quartz sand, subsequently converted the [^11^C]CH_2_O to [^11^C]HCN (Fig. 1). Compared to the traditional method, the yield of [^11^C]HCN was comparable, and the molar activity was three times higher. Thus, with this method, [^11^C]HCN can be easily obtained using readily available lab ware, without the need for a dedicated cyanide synthesizer.

## [^18^F]Flumazenil synthesis through a copper‑mediated ^18^F‑fluorination approach for multi‑patient dose

### By Mohammed Al-Qahtani

Aryl fluoride is present in flumazenil which makes it an advantage and potential to use the radionuclide fluorine-18 Positron Emission Tomography (PET) targeting imaging, without any chemical modification to the parent structure. Several labeling strategies have been reported with general drawbacks such as low radiochemical yields (RCYs), poor stability and availability of certain precursors, and many others. However, the copper-mediated ^18^F-fluorination of aryl boronic species has been applied to a wide variety of structurally diverse targets, including flumazenil (FMZ).

An automated radiosynthesis of [^18^F]FMZ from the corresponding pinacol-boronate ester precursor was recently described (Gendron et al. 2022). The methodology is configured by applying a single cassette-based synthesis of [^18^F]FMZ using the Trasis AllinOne (AIO) module which is fully compatible with Good Manufacturing Practice (GMP). Moreover, short production time and simplified purification procedures, whilst ensuring clinically useful RCYs and a final product quality compliant for human injection are reported.

**Fig. 2:**
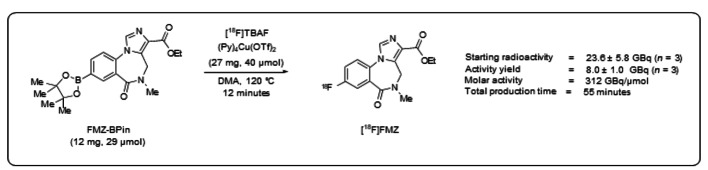
Optimised automation conditions for the production of [^18^F]FMZ (Reproduced with permission (Gendron et al. 2022))

The automated protocol was performed at two different starting activities: using 300–340 MBq (n = 5, Trasis side; Trasis, Rue Gilles Magnee, 90, 4430 Ans, Belgium) of [^18^F]fluoride, this method yielded [^18^F]FMZ in 43 ± 2% n.d.c and using increased starting activities (23.6 ± 5.8 GBq, n = 3, Cardiff side; Wales Research and Diagnostic PET Imaging Centre, Cardiff University, University Hospital of Wales, Heath Park, Cardiff CF14 4XN, UK) of [^18^F]fluoride, this method yielded [^18^F]FMZ in 35 ± 5% n.d.c. The molar activity of [^18^F]FMZ was 312 GBq/µmol n.d.c (Fig. 2). The authors did emphasize that the less basic nature of oxalate in comparison to carbonate was found to be crucial to obtaining sufficient RCYs. Most importantly, the presented fully automated radiosynthesis procedure is proven to be a useful approach for providing multi-patient doses of [^18^F]FMZ.

## A Promising Versatile Chelator for Alpha-Emitting [^203^Pb]Pb^2+^, [^213^Bi]Bi^3+^, and **[**^**225**^**Ac]Ac**^**3+**^

### By Ya-Yao Huang

The development of versatile chelators will be much helpful to increase the utility, usability, and availability of α-emitter labeled bioconjugates used in targeted alpha therapy (TAT). In 2017, an expanded 18-membered macrocyclic MACROPA has been proved as an effective chelating agent for large ion ^225^Ac with excellent stability (Thiele et al. 2017), but its poor affinity and stability in binding smaller ions may hinder its use in TAT (Hu et al. 2021). On the other hand, acyclic chelators based on a pyridinecarboxylate scaffold has shown its utility with an array of metals with medical applications, such as H4octapa (Jaraquemada-Peláez et al. 2017; Platas-Iglesias et al. 2004) and its amido-derivative of hybrid EGTA ligand, ampam (Ingham et al. 2021).

Combined with the concept of MACROPA and ampam, H2ampa was designed (Ingham et al. 2022) and further proven to be a versatile chelator for [^203^Pb]Pb^2+^, [^213^Bi]Bi^3+^, and [ ^225^Ac]Ac^3+^ ions with a favorable chelation conditions, such as short reaction times (7–30 min), at dilute concentrations, and under mild conditions. Furthermore, H2ampa radiolabeled [^203^Pb]Pb^2+^, [^213^Bi]Bi^3+^, and [ ^225^Ac]Ac^3+^ ions generated molar activities of ∼20, ∼372, and ∼130 MBq/µmol respectively. However, further labelling studies and stability tests are warranted in order to assess the radiopharmaceutical potential of H2ampa thoroughly.

## A ^89^Zr-labeled PSMA tracer for PET/CT imaging of prostate cancer patients

### By Emiliano Cazzola

A wide number of prostate-specific membrane antigen (PSMA) tracers were developed in the last years and different radionuclides were use during these development processes. More tailored biological functions were obtained by study the internalization process and protein structure. In this development prospective, different radionuclides were involved like gallium-68, fluorine-18 and technetium-99 m and a large number of radiopharmaceuticals became available for nuclear medicine. Here, Dietlein and co-workers took in consideration the relations between biological interaction (internalization) with the radionuclide half-life used for radiopharmaceutical production (Dietlein et al. 2022). The possibility to scan at later time points, due to the 78.9 h half-life of zirconium-89, opens the way to wait for an increased accumulation in tumor cells, thereby increasing the signal-to-noise ratio. In this connection, ^89^Zr-PSMA-Df, in a small group of patients, obviously was able to localize more positive lesions (8/14), compared to ^68^Ga-PSMA-11 (1(/25) and ^18^F-JK-PSMA-7 (1/10). This study demonstrating that, in patients with weak PSMA expression, a radionuclide with longer half-life (^89^Zr), resulting in a better diagnosis, due to increased internalization. Once again, the biological process needs to be associated to the appropriate radionuclide in terms of half-life, emission, chemistry, biodistribution and radiation exposure evaluation to create the optimal image and as a consequence the beter diagnosis.

## Are nanoparticles suitable vectors for cancer therapy using Auger Electrons emitting radionuclides?

### By Sergio Todde

Due to their inherent radiophysical properties, Auger Electrons (AE) emitting radionuclides are well suited for cancer therapy with radiopharmaceuticals. However, their short range may be a limiting factor, as they need to be delivered close to their biological target (i.e. DNA/RNA) to exert their effect. To this regard, nanoparticles (NPs) may represent a suitable delivery system, especially due to their versatility in functionalization, large surface/volume ratio, and enhanced permeability and retention. A comprehensive review of studies performed was recently published with a wide variety of combination of organic and inorganic nanoparticles functionalized with suitable targeting molecules (e.g. antibodies, peptides), nuclear localization signals and chelators, in most cases radiolabelled with high molar activity with indium-111 or iodine-125 (Gharibkandi et al. 2022). Additionally, in some of the considered works sense/antisense sequences, PEG or other moieties aimed to improve target recognition and cell internalization rate were also part of the whole radiobiological conjugate. Results achieved following in vitro studies are often encouraging, and significant improvement in therapeutic efficacy has been demonstrated, prompting for nanoparticles as a potential added value. However, clinical application in human subjects are still not common, major limiting factors being high retention of the labelled NPs in liver and spleen, with fast blood clearance and poor tumour uptake. However, strategies to overcome the above limitations are continuously refined; positive examples are studies performed with high-Z element nanostructures as sensitizers of external X-ray radiation, which have now moved to the stage of clinical trials, and targeted nanobrachytherapy approaches, that may help to overcome retention in liver and spleen.

## A novel [^18^F]difluorocarbene reagent for versatile ^18^F-difluoromethylation

### By Shozo Furumoto

**Fig. 3:**
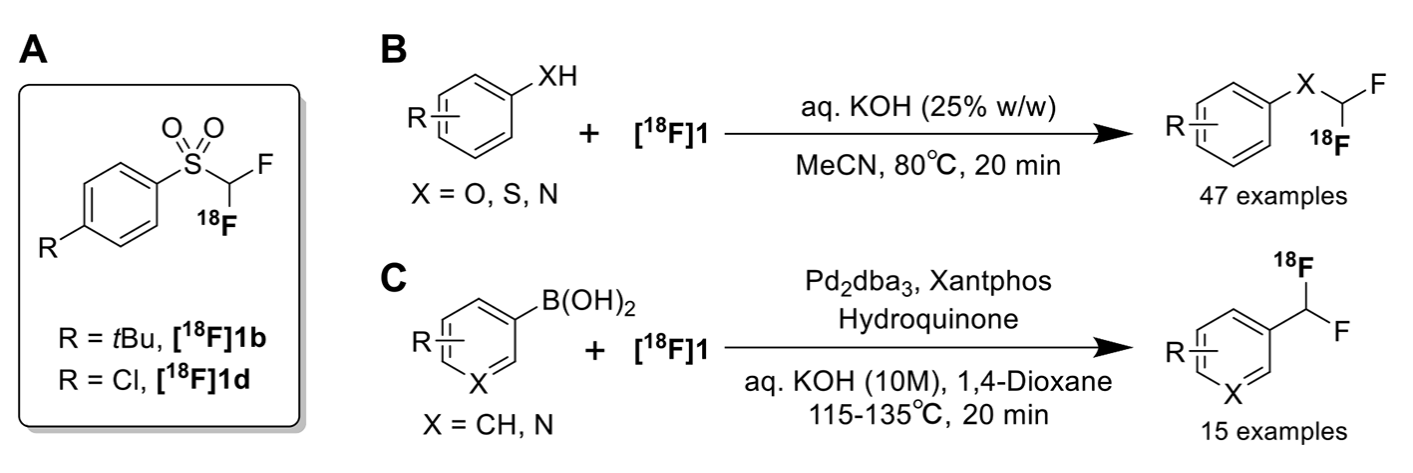
A new [^18^F]difluorocarbene reagent (A) and its application to the ^18^F-difluoromethylation reactions of (thio)phenols and N-heterocycles (B) and aryl boronic acids (C)

Fluorine-18 is one of the most valuable radionuclides for positron emission tomography due to its suitable properties such as excellent positron emission rate (97%), low positron emergy (635 KeV), and favorable half-life (109.9 min). These advantages have led to numerous studies on ^18^F-radiochemistry. Establishing various ^18^F-radiolabeling methods will facilitate molecular design strategies for ^18^F-labeled tracers and expand their structural diversity. Therefore, researchers are still actively investigating new ^18^F-radiolabeling reactions. Recently, a new [^18^F]difluorocarbene reagent **[**^**18**^** F]1** (Fig. 3 A) has been reported available for versatile ^18^F-difluoromethylation as an alternative to ^11^C-methylation (Sap et al. 2022). Using the [^18^F]difluorocarbene reagent, they prepared 47 ^18^F-difluoromethylated derivatives, which include 19 bioactive compounds, from (thio)phenols and N-heterocycles (Fig. 3B). They further achieved the synthesis of ^18^F-difluoromethylated aryl derivatives (15 examples, including five bioactive compounds) by the Pd-catalyzed cross-coupling reaction of the [^18^F]difluorocarbene reagents with aryl boronic acids (Fig. 3 C). To examine the utility of the ^18^F-difluoromethylated tracer as a PET imaging agent, they synthesized a derivative of [^18^F]DPA-714 in which the ^18^F-fluoroethyl group was replaced by the [^18^F]difluoromethyl group. PET imaging study using a mouse model of Huntington’s disease clearly demonstrated that the ^18^F-difluoromethylated derivative of DPA-714 was successfully used to image microglial activation in the striatum. One notable result was the absence of radioactive metabolites in plasma and brain tissue 5 min after administration, suggesting in vivo metabolic stability of the [^18^F]difluoromethyl group. These results will greatly encourage further studies on ^18^F-difluorocarbene chemistry and the development of the ^18^F-difluoromethylated PET tracers.

## Current status on cyclotron facilities – a dynamically changing landscape

### By Renata Mikolajczak

Nowadays the role of cyclotrons for production of medical radionuclides for Positron Emission Tomography (PET) imaging is undeniable. Cyclotrons are a resource for radionuclides for clinically established radiopharmaceuticals for patient care in nuclear medicine and for the development of new tracers for the regional needs. This imaging techniques primarily relies on the availability of cyclotron-produced radionuclides, preferably from the on-site or nearby cyclotron facility within a convenient distance. The focus of the recent report (Avila-Rodriguez et al. 2022) is on the cyclotrons as the production facilities of radiopharmaceuticals in the Latin America and Caribbean region and presents the state-of-the-art in PET radiopharmaceuticals developments. Notably, the first compact cyclotron for PET radionuclide production in Latin America was installed in 1997, in the following years this innovative imaging technique captured the attention of the medical and scientific community and by 2010 over 15 more compact cyclotrons were already installed in Latin America countries. Based on the on-line survey and direct communications, in the current report the information was collected on 67 cyclotrons in operation, in progress of installation and in commissioning, as well as cyclotron projects with signed contracts, both in public and in private sectors. The role of International Atomic Energy Agency (IAEA) in the capacity building, knowledge sharing and promotion of the regional production and use of PET is to be mentioned.

The report is answering the need for consolidated and updated information on the cyclotron infrastructure, which was also recently addressed (Zippel et al. 2022) in Europe for the region of German speaking countries (Germany, Austria and Switzerland). In total, 42 cyclotrons were identified. The vast majority of them are operated by universities, university hospitals or research institutions in close proximity to a university hospital, less by/in cooperation with industrial partners or a non-academic clinic/PET center.

The landscape of cyclotron facilities is changing dynamically (IAEA database on cyclotrons, 2022). The very good news is that the number of actively operated cyclotrons increases. It holds the promise for the increase in nuclear medicine diagnostic and therapeutic procedures for which cyclotron-based radiopharmaceuticals are used.

## Rethinking targeting for radionuclide therapy

### By Clemens Decristoforo

Targeted Radionuclide Therapy (TRT) has gained increasing attraction by the success of theranostics especially with the clinical establishment of ^177^Lu-labelled Somatostatin (SST) analogues and PSMA ligands. More and more research focusses on other radionuclides such as alpha- and Auger electron emitters. Due to their short range a high linear energy transfer is reached with the potential of higher therapeutic efficacy and overcoming resistance phenomena. The current paradigm for Auger electron emitters is based on the necessity for intracellular and nuclear localization to reach a therapeutic effect. A highly interesting radionuclide in this context is terbium-161 with similar nuclear properties as compared to lutetium-177 and almost identical chemistry, but additional high emission of low energy electrons (Auger and conversion).

Preclinical therapeutic studies with SST analogues labelled with terbium-161 and lutetium-177 have been published (Borgna et al. 2022). The agonists DOTA-TOC and NLS-DOTA-TOC (containing a nuclear localizing sequence) were compared with the antagonist DOTA-LM3 regarding their in vitro behaviour in SST receptor expressing cells including subcellular localization, their biodistribution in tumour bearing mice and finally their preclinical therapeutic efficacy. Interestingly, in vitro they found a more than 100-fold higher therapeutic efficacy of terbium-161 vs. lutetium-177 labelled DOTA-LM3, an effect, that was almost not seen with the corresponding SST-agonists, despite their much more efficient nuclear targeting. They could confirm their findings in vivo, where only mice treated with [^161^Tb]Tb-DOTA-LM3 survived the therapeutic study.

This study again showed the suitable properties of terbium-161 as a radionuclide with certain advantages over lutetium-177, but, more importantly, supports the change of paradigm in targeted radionuclide therapy from the necessity of intracellular targeting of short ranged radionuclides (in particular Auger electron emitters) to more effective therapeutic outcome when targeting the cell membrane structures. These results are therefore another important milestone, that confirms findings from other radiobiology studies (Pouget et al. 2022). Consideration of this paradigm shift may be essential in the successful future development of novel radiopharmaceuticals for TRT.

## Strained ammonium precursors for Radiofluorinations

### By Nic Gillings

[^18^F]Fluoroethylamino and [^18^F]fluoropropylamino groups are common in many ^18^F-radiopharmaceuticals, and there are several established methods for their preparation from cyclotron produced [^18^F]fluoride. Such methods often require harsh radiolabelling conditions, which may not be compatible with many biomolecules. Strained ammonium precursors have the potential to facilitate fluorine-18 incorporation in a mild and regioselective manner, to expand the scope for preparation of such moieties. In a short review (Reissig et al., 2022), the authors have presented the current state of the art with respect to the use of 3-membered nitrogen containing rings (aziridines or aziridium salts) and the equivalent 4-membered rings (azetidinium salts) for ^18^F-labelling reactions.

There are a number of examples presented which demonstrate the feasibility of fluorine-18 labelling using aziridines, provided there is a suitable electron-withdrawing-group present on the ring nitrogen. By using in-situ formation of aziridinium salts instead, activation with an electron withdrawing group is not required, and radiolabelling often proceeds efficiently at room temperature. Furthermore, these techniques are also applicable to the preparation of α- and β-amino acids. Finally, more recent results are presented regarding the use of azetidinium salts, which in several cases demonstrated superior radiochemical yields compared with open-chain radiolabelling strategies.

## Targeted PET imaging of tumor-infiltrating T cells in cancer patients using radiolabeled anti-CD8 minibody

### By Min Yang

**Fig. 4:**
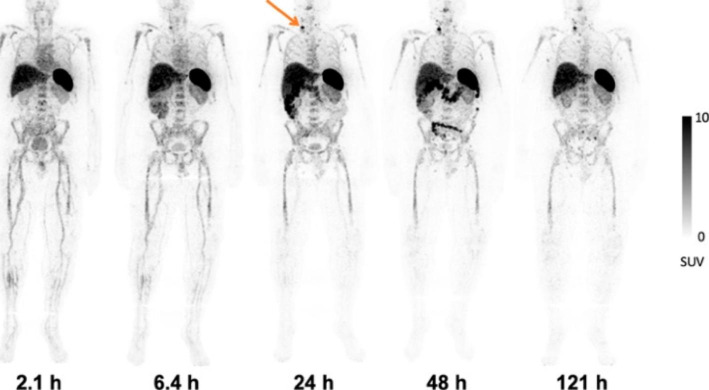
Whole-body PET images of a patient at various times after injection of ^89^Zr-Df-IAB22M2C with good visualization of uptake in the nodal metastasis at 24–48 h post-injection. Reproduced from Farwell et al. (2022)

Immunotherapy has become an important treatment for many malignancies. However, there are still a large number of patients who do not respond well to immunotherapy. Measuring tumor-infiltrating CD8 + leukocytes by in vivo imaging approach can predict early response to cancer immunotherapy and select appropriate treatment regimens. A radiolabeled humanized anti-CD8 minibody, ^89^Zr-Df-IAB22M2C, was developed for PET imaging of tumor-infiltrating CD8 + T cells (Farwell et al. 2022). Fifteen patients with metastatic melanoma, non-small cell lung cancer and hepatocellular carcinoma were involved in this study. ^89^Zr-Df-IAB22M2C was observed to accumulate in tumors and CD8-rich tissues, including spleen, bone marrow, nodules, with maximum uptake 24–48 h post-injection (Fig. 4). ^89^Zr-Df-IAB22M2C uptake in tumors was observed in 10 of 15 subjects, including 7 of 8 subjects who received immunotherapy, 1 of 2 subjects who received targeted therapy, and 2 of 5 subjects who were treatment-naive. Moreover, increased uptake of ^89^Zr-Df-IAB22M2C in tumor lesions was associated with response in three cases with clinical follow-up. Overall, the first-in-humans study of ^89^Zr-Df-IAB22M2C demonstrated its safety and potential to visualize whole-body CD8 + leukocyte biodistribution for further early prediction of the response to immunotherapy.

## Thinking small – radiolabeled nanoparticles for cancer imaging and treatment

### By Raymond M. Reilly

Radiolabeled nanoparticles (NPs) for cancer imaging or treatment were reviewed (Pijeira et al. 2022). NPs are drug delivery systems for cancer treatment with dimensions of 1-100 nm (Kinnear et al. 2017). NPs may accumulate in tumours by the enhanced permeability and retention (EPR) effect or be targeted to tumours by modification with antibodies, peptides or aptamers. A major challenge is sequestration of NPs by the liver and spleen, which is minimized by coating with polyethyleneglycol (PEG). ^64^Cu-labeled NPs (e.g. liposomes or polymeric micelles) imaged prostate cancer, breast cancer, lung cancer and other tumours in mice by PET. Tumour uptake was mediated by the EPR effect or by targeting epidermal growth factor receptors (EGFR), bombesin receptors, α_v_β_3_ integrins or PD-1. In one study, PET with ^64^Cu-labeled NPs probed the EPR characteristics of tumours in mice, which was directly correlated with response to drug-loaded NPs (Lee et al. 2018). These ^64^Cu-NPs were also studied in patients with HER2-positive breast cancer to assess tumour EPR and predict treatment response (Lee et al. 2017). The application of radiolabeled NPs for cancer therapy is largely unexplored. Most studies have focused on intratumourally injected gold NPs labeled with the β-particle emitter, ^177^Lu. These strongly inhibited tumour growth in mice with minimal normal tissue toxicity (Cai et al. 2017). Loco-regional administration of NPs may be feasible for anatomically confined tumours (e.g. GBM). Preclinical studies of NPs labeled with ^186^Re, ^188^Re or ^177^Lu infused locally by convection enhanced delivery have shown promise for treatment of GBM (Phillips et al. 2014). Intratumoural delivery under image guidance for treatment of oligometastatic disease may also be possible. There are opportunities in the future to radiolabel NPs with powerful α-particle emitters, such as ^223^Ra or ^225^Ac for cancer treatment.

## Imaging macrophages to Chase Up Cancer

### By Adriano Duatti

Despite the considerable success of receptor radionuclide therapy (RRT), it is also necessary to recognize that, based on the results of ongoing clinical studies, this approach is revealing some intrinsic limitations. A major drawback is that receptor expression is a dynamic process that could be strongly affected by a variety of factors including the RRT procedure itself. For instance, downregulation of receptor expression may occur after internalization of the radiopharmaceutical with the consequent decrease of the number of receptors available for tumor targeting. Evidently, the search for other targets less prone to tumor genetic instability and variability is becoming a broad field of investigation.

Tumor microenvironment (TME) is a very attractive, alternative target for radionuclide therapy and tumor-associated macrophages (TAMs) play a crucial role in building up TME. Since TAMs can represent approximately 30–50% of the tumor mass and trigger several factors that promote tumor growth and metastasis, they have been considered as potential targets for early cancer detection and treatment and TAM-targeted therapies is currently actively explored.

Considering the growing importance of TAMs as an alternative strategy for future targeted radionuclide therapy, the article entitled ‘Potential PET tracers for imaging of tumor‑associated macrophages’ (Fernandes et al. 2022) is of significant value as it provides an excellent overview of the status of the TAM biomarkers, for which potential PET-tracers are already available and under active preclinical and clinical evaluation.

## OncoFAP just as good or even better than FAPI-46?

### By Antonia Denkova

**Fig. 5:**
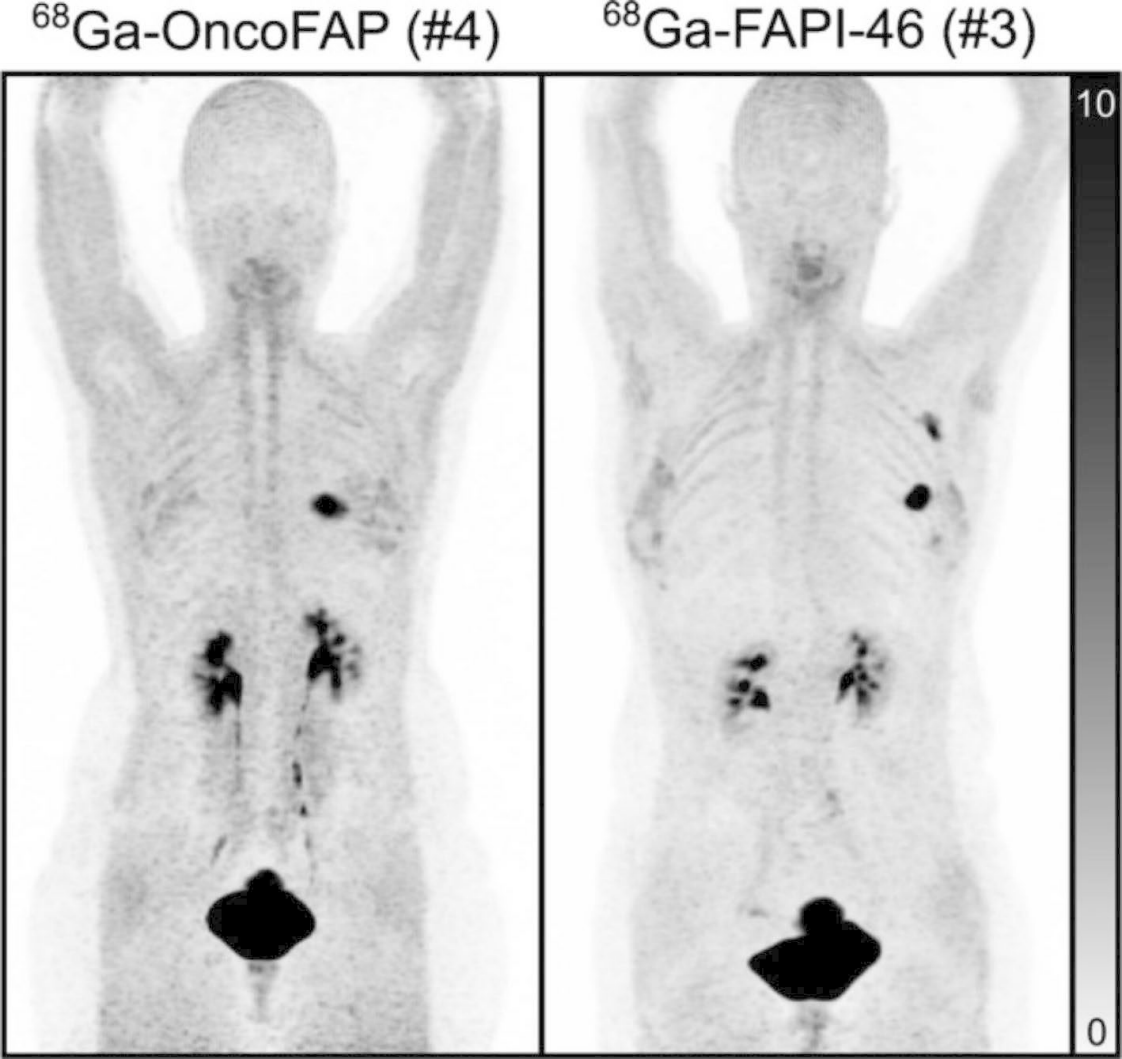
Comparison between OncoFAP left and FAP-46 right, for two different patients both having breast cancer. The images show very similar behaviour of the tracers (with permission from Backhaus et al. 2022)

The fibroblast activation protein (FAP) has drawn a lot of attention in the last years due to its great promise as a targeting agent for both cancer diagnostics and radionuclide therapy. A derivative of FAP, OncoFAP has recently been developed showing improved affinity for malignant tissue (Millul et al. 2021). The paper ‘Translational Imaging of the Fibroblast activation protein (FAP) using the new ligand [^68^Ga]Ga-OncoFAP-DOTAGA highlights the first clinical trials and demonstrate its potential (Backhaus et al. 2022). The authors first performed pre-clinical studies in murine tumour models expressing FAP which showed better uptake 1 hour post injection when compared to the developed by the University of Heidelberg FAPI-46 ligand, but with similar washout characteristics. Moreover, the pharmacokinetic modelling suggested that the higher uptake might be due to higher affinity of OncoFAP, although at 3 hours post-injection the uptake levels between the two ligands were not significantly different. In human patients OncoFAP performed equally well as FAPI-46 demonstrating high uptake in the primary tumours, lymph node metastases and distant metastases (Fig. 5). At the same time the new FAP ligand appeared to be taken somewhat less in the liver than FAPI-46. In conclusion, this paper proposes a new promising FAP ligand for future clinical studies.

## Imaging of cerebral tryptophan metabolism using 7-[^18^F]FTrp-PET in a unilateral parkinsonian rat model

### By Ralf Schirrmacher

Molecular imaging and quantification of the human tryptophan (Trp) metabolism could help answering important questions associated with the progression and severity of Parkinson’s Disease (PD). Patients afflicted by PD show a maladjustment with regard to serotonin production, one major hallmark of PD, and an enhanced flare-up of neurotoxin production stemming from the kynurenine metabolic pathway. A third pathway capitalizes on the metabolism of Trp to serotonin and finally melatonin. All three processes originate from Trp as the common precursor. To investigate Trp metabolism in PD, only the ^11^C-tracer α-[^11^C]methyl-l-tryptophan has been used so far, limiting studies to PET centers possessing an on-site cyclotron. Neumaier and co-workers reported on the synthesis of 7-[^18^F]fluorotryptophan ([^18^F]FTrp), a PET tracer to investigate all three metabolic Trp pathways facilitating this line of PD research (Endepols et al. 2022). [^18^F]FTrp is a more readily available radiotracer which allows satellite production and permits longer imaging protocols and improved logistics. When injected into 6-OHDA rats, a common rat model for PD characterized by the unilateral injection of the neurotoxin 6-hydroxydopamine inducing parkinsonism, [^18^F]FTrp depicted changes in all three Trp metabolic pathways. Reduced serotonin synthesis in conjunction with increased melantonin production in the pineal gland in 6-OHDA rats was observed, rendering this new tracer capable of detecting molecular changes in PD related neurodegeneration. Hopefully a human clinical translation will follow soon to consolidate the promising pre-clinical data.

## FAPi dimerization: a potential approach for tumor theranostics

### By Giuseppe Carlucci

Cancer-associated fibroblasts (CAFs) are crucial components of the tumor microenvironment. Fibroblast activation protein (FAP) is overexpressed in the CAFs in numerous epithelial carcinomas and weakly expressed in healthy tissues; therefore, FAP represents an attractive target for theranostics. This study aims at exploring a dimeric FAPi compound in a human translational setting. The goal is to enhance the tumor-targeting efficacy, uptake and retention of FAPi. Specifically, DOTA-2P(FAPi)_2_, a FAPi dimer was designed and synthesized with two mini-PEG spacers (11-amino-3,6,9-trioxaundecanoic acid, with three ethylene oxide units) between the two FAPi motifs (Zhao et al. 2022). DOTA-2P(FAPI)_2_ was labeled with ^68^Ga ([^68^Ga]Ga-DOTA-2P(FAPI)_2_) and tested in PDX models, healthy volunteers, and cancer patients. [^68^Ga-DOTA-2P(FAPI)_2_ exhibited improved in vivo pharmacokinetics and enhanced tumor uptake compared to the monomer. HCC-PDX groups showed prominent tumor uptake and predominant organ clearance. The success of the preclinical studies led to the clinical translation of [^68^Ga]Ga-DOTA-2P(FAPI)_2_ into human subjects. [^68^Ga]Ga-DOTA-2P(FAPI)_2_ showed a rapid and stable accumulation in tumor lesions in both mouse models and humans. The retention of the tracer in the patient blood pool remained high 4 h p.i. with an average 0.0119 mSv/MBq effective whole-body dose. Multimerization remains an important tool to improve targeting of peptide radiopharmaceuticals. The consistent higher tumor uptake of multimers render them potentially superior to monomers and make them interesting platforms for applications where high tumor accumulation is crucial, such as for radiotherapeutics.

## Will FAP-targeted peptides outclass the current clinical small-molecule FAP inhibitors?

### By Yann Seimbille

Development of fibroblast activation protein (FAP) targeted small molecule-based radiopharmaceuticals has gained a tremendous attention over the last few years. A plethora of radiolabeled FAP inhibitors has been discovered, and among them few compounds, such as FAPI-04, FAPI-46, FAPI-74, QCP02, RPS-309 and OncoFAP are being clinically tested. Although they showed very promising results for cancer imaging, their relatively low tumor retention is a concern for targeted radionuclide therapy. To address this issue, the preclinical evaluation of a cyclic peptide, namely FAP-2286, which was labeled with gallium-68 and indium-111 for imaging and lutetium-177 for therapeutic use was recently reported (Zboralski et al. 2022). The authors anticipated that a cyclic peptide targeting FAP would have an improved tumor retention time over the small molecule-based radiopharmaceuticals. It was indeed confirmed that [^177^Lu]Lu-FAP-2286 had an extended tumor retention compared to [^177^Lu]Lu-FAPI-46 in a FAP-positive tumor bearing mouse model. The radiolabeled peptide was cleared via renal excretion and no other non-target tissues accumulation was observed. Moreover, the absorbed dose delivered to the tumors was 9-fold higher with [^177^Lu]Lu-FAP-2286 than FAPI-46. Taken together, it seems that the FAP targeted cyclic peptide FAP-2286 is outperforming the current small molecule-based inhibitors, such as FAPI-46, but new generation of compounds with improved pharmacokinetic profile, mainly bivalent inhibitors, are currently being developed.

## Dual SPECT and optical imaging with a single agent for image-guided cytoreductive surgery of colorectal cancer metastases

### By Zhaofei Liu

Each imaging modality has its own advantages and disadvantages, and thus the combination of two or more modalities may be needed to provide complementary information for improved patient care. Dual nuclear (PET or SPECT) and optical imaging may be possible using a single agent labeled with a radionuclide and a near-infrared fluorophore. Whole-body localization of tumor burden may be determined by preoperative SPECT or PET imaging, and the lesions could then be precisely resected during surgery under the guidance of intraoperative radio-detection and optical imaging. A phase I clinical trial evaluating an anti-carcinoembryonic antigen (anti-CEA) antibody with dual ^111^In and fluorophore labeling, [^111^In]In-DOTA-labetuzumab-IRDye800CW, for preoperative imaging and intraoperative image-guided cytoreductive surgery of colorectal peritoneal metastases was reported (de Gooyer et al. 2022). [^111^In]In-DOTA-labetuzumab-IRDye800CW is reported to be safe, and this dual-modality imaging strategy is feasible for clinical studies. Moreover, previously undetected metastatic lesions can be identified with this approach, leading to a modification of the clinical strategy based on more accurate information. Upon further optimization (e.g. reducing the liver uptake, improving the imaging contrast, and minimizing the radiation dose), dual radionuclide and fluorophore-labeled imaging with a single agent may have broad clinical applications for a variety of cancer types to guide clinical decision making and improve cancer management.

## [^99m^Tc]Tc-labeled EpCAM targeted nanobody for EpCAM expression imaging

### By Beverley Ellis

**Fig. 6:**
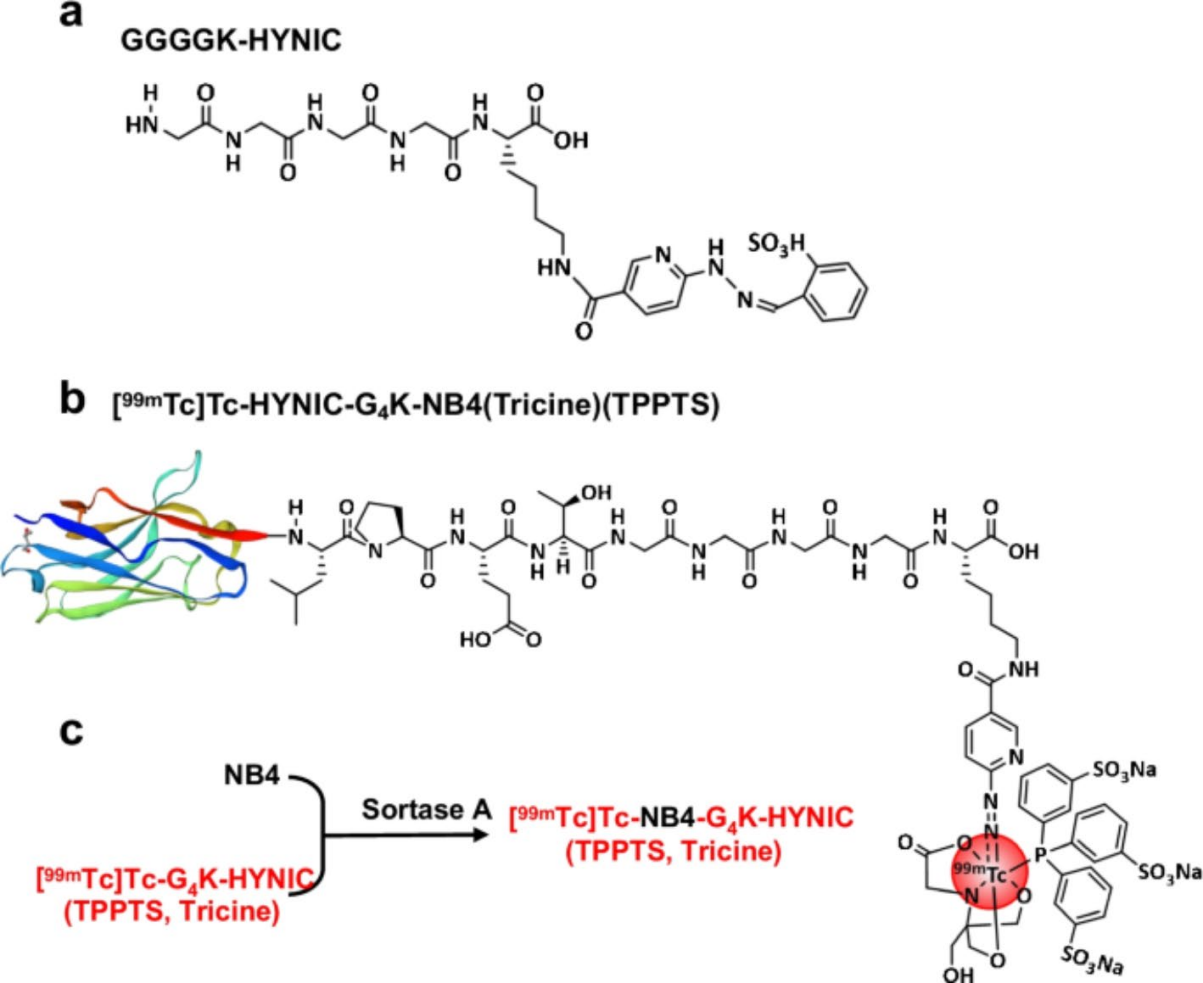
**a**, **b**, Structure of GGGGK-HYNIC and [^99m^Tc]Tc-NB4. **c** Synthetic route of [^99m^Tc]Tc-NB4 (with permission from Liu et al., 2022)

Overexpression of the epithelial cell adhesion molecule (EpCAM) is found on a variety of human adenocarcinomas and squamous cell carcinomas. Molecular imaging agents that target EpCAM would be potentially useful in the detection of epithelium-derived tumours. Nanobody-based molecular probes are reported to have better tissue permeability and faster body clearance compared to EpCAM targeted antibody probes. Recently a new radiolabeled EpCAM targeted nanobody [^99m^Tc]Tc-NB4 was prepared (Lui et al. 2022) (Fig. 6) and the radiochemical purity determined by iTLC was > 97%. In vitro characteristics were investigated in HT-29 (EpCAM positive) and HL-60 (EpCAM negative) cells. Protein binding experiments showed that NB4 bound almost exclusively to the human EpCAM protein and [^99m^Tc]Tc-NB4 displayed a high specificity in cell-binding assays. Pre-clinical SPECT/CT imaging indicated a rapid accumulation and relatively high uptake in subcutaneous EpCAM-positive HT-29 tumours but EpCAM-negative HL-60 tumours could not be imaged. Although the HT-29 tumour uptake increased with time, a high renal uptake was also observed as [^99m^Tc]Tc-NB4 is excreted by the kidneys. SPECT/CT imaging in a lymph node metastasis model could locate small lymph node metastasis tumours indicating a high sensitivity of the probe. The authors conclude that [^99m^Tc]Tc-NB4 has potential for clinical translation as a broad-spectrum SPECT radiotracer for imaging EpCAM expression in epithelium-derived cancer.

## New hypoxia tracer!

### By Bart Cornelissen

PET imaging of tumour hypoxia, the lack of oxygen in tissue, is a hallmark of cancer. It has long been a target of interest for molecular imaging. Hypoxia influences resistance to therapies by inducing cell cycle arrest (quiescence), by inhibiting apoptosis and senescence, by controlling autophagy, p53 activity, and mitochondrial activity. Radiotherapy is far less effective in hypoxic cancerous tissue, due to a lack of reactive oxygen species that can be formed. An ability to quantify hypoxia non-invasively, using PET, would significantly change our ability to serve cancer patients. Nitroimidazole-based compounds like [^18^F]FMISO, [^18^F]FAZA, or [^18^F]HX4 generally suffer from a lack of target-to-background contrast, [^64^Cu]Cu-ATSM does not correlate with hypoxia in vivo, and [^18^F]EF5 requires convoluted synthesis with ^18^F_2_ gas (Gerard et al. 2019).

In their manuscript, Nario et al. report on a novel hypoxia tracer, based on an ^18^F-labelled benznidazole, dubbed [^18^F]FBNA (Nario et al. 2022). Non-radiolabelled benznidazoles may be used as an hypoxia-activated cytotoxic drug. Radiolabelled [^18^F]FBNA is more lipophilic than [^18^F]EF-5, [^18^F]FMISO and [^18^F]FAZA, and is retained in hypoxic cancer cells. Although no in vivo studies were performed, in vitro behaviour showed superlative contrast between hypoxic and non-hypoxic cells, compared to [^18^F]FAZA.

The study highlights the continued search for molecular imaging agents to visual hypoxia, and other hallmarks of cancer. In the spirit of ‘seeing is believing’, visualisation of tumour biology will continue to benefit cancer patients, by enabling the study of cancer biology, therapy selection, adaptive therapy, and measurement of therapy response.

## Can we increase efficiacy of targeted endoradiotherapy through neoadjuvant epigenetic treatment? A new therapeutic strategy

### By Klaus Kopka

**Fig. 7:**
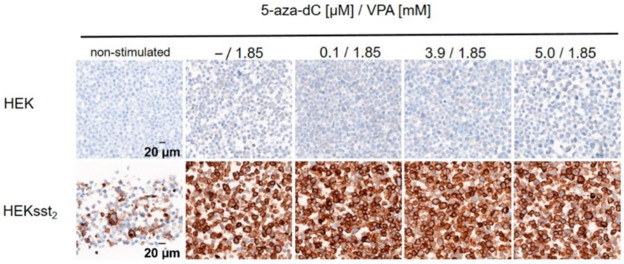
Representative image of SST_2_ receptor staining for HEK and HEKsst2 cells after stimulation by increasing concentrations of 5-aza-dC and constant concentrations of VPA. Non-stimulated HEKsst2 and HEK cells are shown as a reference (40× magnification). The figure is reproduced with permission from Kotzerke et al. (2022)

**Fig. 8:**
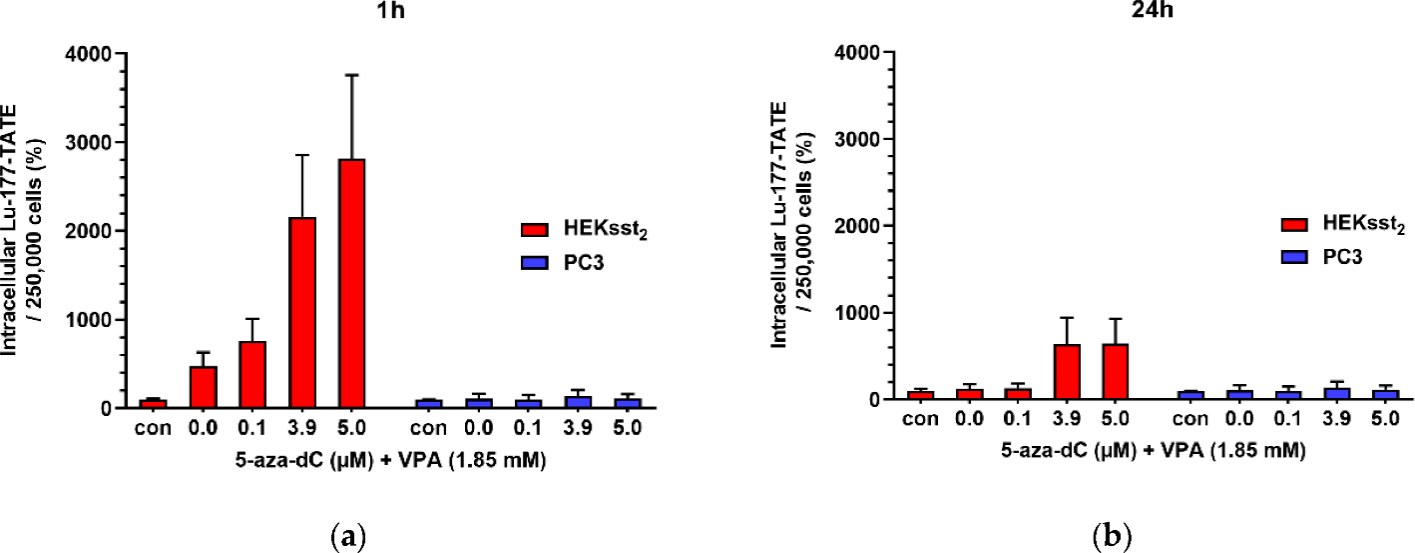
(**a**) Cellular uptake of [^177^Lu]Lu-DOTA-TATE (50 kBq) for 1 h in HEKsst2 and PC3 cells and (**b**) 24 h. Before uptake analysis both cell lines were pretreated with 5-aza-dC and VPA over a time period of 5 days as described above. Unstimulated controls (con) were simultaneously incubated with [^177^Lu]Lu-DOTA-TATE. The intracellular activity is normalized to results in unstimulated control cells. Results are expressed as changes in percentage in relation to results without stimulation. All data are shown as mean ± SD. The figure is reproduced with permission (Kotzerke et al. 2022)

This original basic (preclinical) research article (Kotzerke et al. 2022) aimed at enhancing the uptake of the recently approved SST_2_-targeting peptide radioligand [^177^Lu]Lu-DOTA-TATE by neoadjuvant use of so called epigenetic modifiers (“epidrugs”), i.e. the DNA methyltransferase inhibitor (DNMTi) 5-aza-2’-deoxycytidine (5-aza-dC) and the histone deacetylase inhibitor (HDACi) valproic acid (VPA), a putative radiosensitizer. The hypothesis is to reactivate epigenetically deactivated tumor-suppressing genes by “epidrugs”. The concept within the manuscript was to examine [^177^Lu]Lu-DOTA-TATE uptake (57.5 or 136 kBq/mL) into and treatment response of transduced SST_2_-positive HEKsst2 cells after preincubating the cells with increasing concentrations of 5-aza-dC (0.0, 0.1, 3.9, 5.0 µM) and at the same time constant concentration of VPA (1.85 mM) over five days. For comparison external radiotherapy using X-rays (0.6 or 1.2 Gy) substituting [^177^Lu]Lu-DOTA-TATE was used as a control. Moreover, unstimulated HEKsst2 cells and PC-3 cells (PC-3 cells exhibit only a minimal expression of SST_2_ on the cell surface) were used under the same setup (Fig. 7). The authors showed nicely that “in the case of stimulated HEKsst2 cells, the uptake of [^177^Lu]Lu-DOTA-TATE increased by a factor of 28 in comparison to the unstimulated cells (Fig. 8). Further, stimulated HEKsst2 cells demonstrated lower survival fractions (factor 4)” after [^177^Lu]Lu-DOTA-TATE treatment. If the clinical transfer of this combinational concept is possible, another integrated treatment option would be available in the future for those patients who respond only poorly to peptide radionuclide therapy (PRRT). Targeted molecular radiotherapy of patients suffering (in this case) from neuroendocrine tumors could be realized which show poor differentiation and thus downregulation of SST_2_ during tumor progression and metastases.

## Moving forward while looking back

### By Emerson Bernardes

Radiopharmaceuticals targeting PSMA and GRPR are currently under clinical trials and are being considered as an alternative imaging tool for definitive diagnosis and grading of prostate cancer. In their work, Qiu showed that targeted biopsies provided by the combinatorial PET/CT image of PSMA and GRPR PET/CT tracers significantly improved the diagnosis of prostate cancer patients (Qiu et al. 2022). Although larger studies are still needed, this study provides strong evidence that a combination of targets will offer better care for patients than an imaging agent alone.

**Fig. 9:**
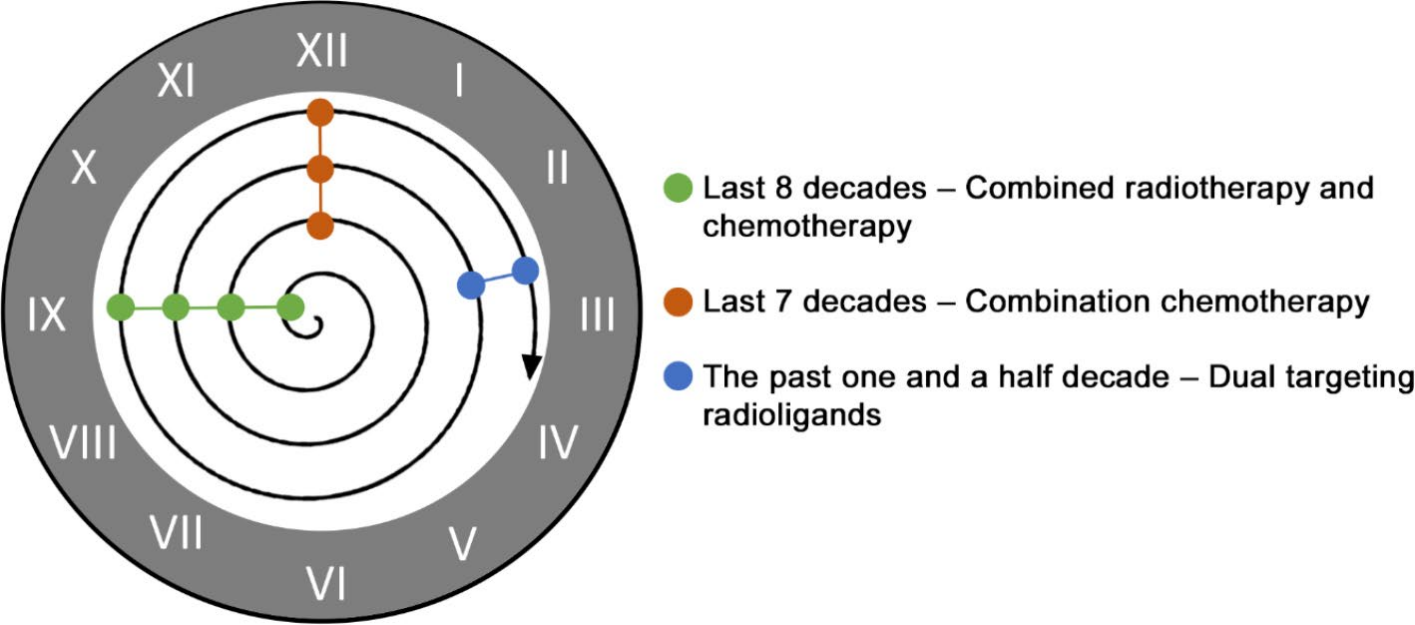
Over the last 8 decades several combinatorial strategies have been repeatedly studied for the management of cancer and will continue being improved by the emergence of new chemotherapeutic drugs, precision medicine technology, lead targets and ligands

Indeed, the combination of either targets or approaches for the diagnosis and treatment of cancer is not novel and has been subject of several research repeatedly over decades including: dual targeting (bispecific tracers, combination of two tracers); combination therapies (also using immunotherapeutic agents); combination chemotherapy ; association of chemotherapy and radiotherapy (Fig. 9). Although it will be challenging to find the right combination of targets and approaches - Cancer is a group of more than 100 distinct diseases, comprised by much wider repertoire of receptors - the pathway to turning cancer into a treatable and manageable condition will include a combination of multiple strategies. Combination of approaches, combination of targets: Welcome back to the future!

## Conclusion

Trends in radiochemistry and radiopharmacy are highlighted demonstrating the progress in the research field being the scope of EJNMMI Radiopharmacy and Chemistry.

## Data Availability

Datasets mentioned in this article can be found in the cited articles.

## Abbreviations

AE: auger electron.
Bq: Becquerel.
CAF: cancer-associated Fibroblasts.
FAP: fibroblast activation protein.
FMZ: flumazenil.
GRPR: gastrin-releasing peptide receptor.
IAEA: International Atomic Energy Agency.
NP: nanoparticle.
PCa: prostate cancer.
PET: positron emission tomography.
PSMA: prostate-specific membrane antigen.
RCY: radiochemical yield.
SPECT: single photon emission computed tomography.
SSTR: somatostatin receptor.
TAM: tumor associated Macrophages.
TAT: targeted alpha therapy.
TME: tumor micro environment.
TRT: targeted radionuclide therapy.
